# The Influence of Odors on Time Perception

**DOI:** 10.3389/fpsyg.2016.00181

**Published:** 2016-02-17

**Authors:** Jean-Louis Millot, Lucie Laurent, Laurence Casini

**Affiliations:** ^1^Laboratoire de Neurosciences Intégratives et Cliniques (EA 481), Université de Franche-ComtéBesançon, France; ^2^Laboratoire de Neurosciences Cognitives-UMR 7291, Université d’Aix-MarseilleMarseille, France

**Keywords:** odor, timing, emotion, hedonic valence

## Abstract

The effect of an olfactory stimulation on the perception of time was investigated through two different experiments based on temporal bisection tasks. In experiment 1, the durations to be classified as either short or long were centered on 400 ms while in Experiment 2 there were centered on 2000 ms. The participants were different in the two experiments (36 subjects in each one). In each experiment, half of the subjects learnt the anchor durations when smelling an unpleasant odor (decanoic acid) and the other half when smelling no odor. After the learning phase, both groups were tested with and without odor. The results showed opposite effects depending on the duration range. The subjects underestimated the time in the presence of the unpleasant odor in the short duration range while they overestimated it in the long duration range. The results have been discussed in the framework of the pacemaker-counter clock model and a potential emotional effect induced by the odor on the subjective time perception has also been considered.

## Introduction

Abilities of human beings to accurately estimate durations are well established. For many years, the prevalent guiding theoretical framework for understanding how we measure the duration of intervals has proposed that we time intervals using an internal clock functioning as a stopwatch, with a clock stage composed of a pacemaker-counter device (Gibbon, 1977; Gibbon et al., 1984). An interval is specified by the accumulation of pulses emitted at a regular rate from a pacemaker. The more pulses that are accumulated, the longer the subjective estimation of duration is.

Nevertheless, this subjective duration of time can be more or less different from the actual duration. The influence of different factors on this internal clock has been extensively studied. The two most documented effects are that subjective duration depends on attention allocated to time (for reviews: Hicks et al., 1976, 1977; Macar et al., 1994; Brown, 1997; Casini and Macar, 1997; Burle and Casini, 2001), and arousal level (Treisman et al., 1990, 1992; Penton-Voak et al., 1996; Burle and Casini, 2001). It has been proposed that arousal level would affect the pacemaker rate. An increasing level of arousal would speed up the pacemaker rate resulting in a larger amount of accumulated pulses and therefore in overestimated durations. On the other hand, attention would affect the accumulation of pulses. Each time attentional resources are diverted from the temporal parameters, pulses are lost, reducing the number of pulses accumulated, and yielding shorter estimated durations. Conversely, if more attention is paid to the duration, more pulses will be accumulated and duration will be judged as longer.

This last decade, a growing literature has addressed the question of the influence of emotion on the perception of time (for reviews, see Droit-Volet and Meck, 2007; Droit-Volet, 2013). Several studies have indicated that negative emotions, at least with high-arousal features, induce longer time estimations than neutral affective states. Nonetheless, it has not yet clearly been established whether this effect is due to arousal or attentional effects (Angrilli et al., 1997; Bar-Haim et al., 2010). In these studies, subjects were required to estimate the duration of exposure to emotional stimuli such as pictures, emotional mimicry, or sounds (Gil and Droit-Volet, 2011; Grommet et al., 2011; Mella et al., 2011). A main problem raised by most of these studies is that the characteristics of the emotional stimuli themselves (for example, number, intensity...) may also affect time perception, independently of the induced emotional state (Droit-Volet, 2013). Thus this type of experiment does not allow for the accurate distinction between arousal and attentional effects because the stimulus which attracts attention is also the same one estimated for its duration. Indeed, time overestimations observed with stimuli inducing negative emotions could then be explained by an increase of either arousal or attention levels, both effects providing similar results. Effects of emotion *per se* have also been studied by comparison between sadness, fear, and neutral mood induced by films shown before a temporal bisection task (Droit-Volet et al., 2011) or between fear (induced by an electric shock) and neutral state (Fayolle et al., 2015). The results have shown that the feeling of fear lengthened time perception.

Considering this background, the originality of the present study is to use an olfactory stimulus as an external factor and to investigate its effect on temporal judgments of neutral stimuli (i.e., sounds). Odors can readily influence emotional states in different situations with little cognitive mediations (Rouby and Bensafi, 2002; Millot, 2009; for reviews). Indeed, hedonic valence appears as the most immediate and important perceived feature of any olfactory stimulation (Alaoui-Ismaïli et al., 1997; Millot and Brand, 2001; Bensafi et al., 2002a). Reviewing the literature, there is only one previous study on time perception using odors as an external factor. Schreuder et al. (2014) used ambient odors to modulate the arousal states of the subjects. Participants had to produce three time intervals (1.33, 1.58, and 2.17 min) when they were exposed to either an arousing odor (rosemary), a relaxing odor (peppermint) or no odor (control condition). When participants were exposed to rosemary odor, they produced significantly shorter intervals than in the no odor condition. Therefore, this effect could not be explained by an increase of arousal but rather by other effects due to odor exposure. It could be noted that the odors used in this study were both judged as pleasant by the subjects.

In the present study, we used decanoic acid as odor, first, because this odor stimulates only the olfactory sense but not the trigeminal nerve (Doty et al., 1978), and second because this odor is judged as slightly unpleasant, often compared to goat odor (Weierstall and Pause, 2012), which should be more appropriate to influence time perception since more data reported an effect of negative emotion on time perception (Angrilli et al., 1997; Droit-Volet et al., 2013).

Participants were required to perform a temporal bisection task in which they were initially trained to discriminate between a short and long duration signal—the anchor durations. In the subsequent test phase, they classified probe signals as short or long, relative to the anchor durations experienced in training. Some of these probe signals were the same as the anchor durations, but most were of intermediate duration. This task has the advantage of providing two distinct measures of performance: the difference limen (DL), which can be interpreted as a measure of participants’ temporal precision, and the point of subjective equality (PSE), which determines whether or not participants presented a shift in their temporal judgments with either an underestimation or an overestimation of durations. These two indices have been classically used to examine effects of attention, memory, and pacemaker changes in interval timing. If the unpleasant odor increases arousal level, participants should overestimate duration and, conversely, if the unpleasant odor captures attention, less attentional resources are available for the stimulus to be judged and participants should therefore underestimate duration.

Moreover, in the field of the psychology of time, a distinction is often made between the processing of durations superior or inferior to one second. Some authors propose that time estimation of hundreds of milliseconds to seconds (supra-seconds durations) would be cognitively mediated whereas measurement of tens to a few hundreds of milliseconds (sub-second durations) is supposed to be of a highly perceptual nature and not accessible to cognitive control (Michon, 1985; Rammsayer and Lima, 1991; Karmakar and Buonomano, 2007). However, some behavioral data also suggest that common mechanisms are involved for both short and long durations (Rammsayer and Ulrich, 2005; Casini et al., 2013). As a consequence, the issue of timescale specificity is still debated and it appears relevant to check whether a negative emotion induced by odor affects the two duration ranges in a similar manner. To tackle this question, two different experiments were performed, the first one used short range durations centered around 400 ms and the second one used long range durations centered around 2000 ms. In each experiment, half of the subjects were trained without odor and tested without then with odor and the other half were trained with odor, then tested with then without odor.

In addition, due to the scalar property characteristic of temporal processing, an effect on the pacemaker rate should be multiplicative with the duration values (Penney et al., 2000; Burle and Casini, 2001). Indeed, if the pacemaker runs faster, the effect has to be greater for longer than for shorter durations (i.e., proportional to the duration values). Using two different duration ranges should therefore also help us to more precisely understand the way how odor modulates time estimation.

## Experiment 1

### Materials and Methods

#### Participants

Thirty-six female undergraduate students (age 18–29 years, mean age = 21.44, SD = 2.1) from the University of Franche-Comté in Besançon (France) were enrolled in this study. Only women were included as there is an asymmetry in olfactory perception in favor of females (Brand and Millot, 2001). All participants were free of nasal allergies and/or head colds. They all gave written informed consent to the experimental procedure, following the Helsinki Declaration (1964). The study was approved by the local ethics committee (CPP Est II).

#### Material and Procedure

The participants were comfortably sat in a quiet, well-ventilated room facing the 15 ″screen of a computer on which instructions were delivered along the experiment. Sounds (white noise) were delivered through headphones and responses were given by using keys A or P of the keyboard. The experiment was controlled by a computer running T-scope (Stevens et al., 2006).

Subjects were aware that the experiment concerned the influence of odors on time perception. The task was to judge the duration of a sound and consisted of three phases: a training phase and two test phases. The total duration of the experiment was about 15 min.

The training phase consisted of two parts. First, participants were presented with the two standard durations (208 and 592 ms), each presented five times in alternation. Participants were instructed just to listen to the stimuli with no response required. Next, the two anchor durations were randomly presented ten times and subjects had to classify them as “short” or “long” by pressing the appropriate response key. The assignment of the keys to the short and long duration was counterbalanced between participants and maintained for the whole experiment. Feedback was not given after each response but only at the end of the block of ten trials, as in the test phase. If the percentage of correct response was inferior to 70%, subjects performed the whole training phase again; otherwise they performed the two test phases.

In each of the two test phases, sounds could be of five different durations (208, 304, 400, 496, 592 ms). Participants were required to indicate whether the presented stimuli were short or long by pressing the appropriate response key. Feedback was not given. Each test phase contained one block of fifty trials corresponding to five stimuli (=5 durations), each delivered ten times (inter-trial interval = 2 s). The only difference between the two test phases was that subjects wore a dust and scratch mask soaked either with 1ml of pure decanoic acid (Sigma–Aldrich) or with 1 ml of diethylphtalate, an odorless diluent (Sigma–Aldrich). The choice of 1 ml of decanoic acid was done following preliminary tests on a panel of naïve subjects to obtain an obvious perception of the odor but without real inconvenience. Half of the subjects performed the training and the first test with an odorless mask and the second test with an odorized mask (Group A). The other half of subjects (Group B) performed the training and the first test with an odorized mask and the mask was changed for the no-odor condition in the second test phase. The delay between the two test phases was less than one minute. The subjects were randomly assigned to Group A or B.

At the end of the whole experiment, participants were asked to give a self-rating of intensity and hedonic valence of the odor on linear scales graduated from 0 (low intensity, displeasure) to 10 (high intensity, pleasure).

### Results

#### Self-Ratings of Odor

The subjects gave the odor of decanoic acid a mean rating of 5.94 (*SD* = 1.5) for the perceived intensity and 2.95 (*SD* = 1.84) for the perceived hedonic valence.

#### Temporal Task Results

The classification data obtained in the duration bisection procedure may be quantified as the proportion of long responses the participant made at each sound duration and can be well described by a sigmoidal function. From this psychophysical function, two dependent variables were estimated: the PSE, the DL. There are different ways of calculating the PSE (Wearden and Ferrara, 1995) but they generally yield similar results. Here, we used the linear regression method which is largely employed (Wearden, 1991) to derive slope and intercept parameters and these were used to calculate the PSE. Linear regression was calculated on all points of each individual psychometric function. All regressions produced *r*^2^ values of at least 0.9. The PSE is the signal duration at which a participant is equally likely to classify the signal as short or long. It represents the subjective midpoint between the short and long anchor values the participant learned in training. An increase in the PSE (a rightward shift of the curve) means that participants chose more often to respond “short”; inversely a decrease in the PSE (a leftward shift of the curve) means that participants were biased towards classifying the signal as “long”. The PSE, reflecting a shift of the curve, therefore allows us to observe whether the participants presented a bias in their temporal judgments towards either a shortening or a lengthening of durations. The DL is a measure of the ‘slope’ of the participants’ response function when plotted. It is calculated from the regression line and corresponds to the half difference between the duration the participant classifies as long 25% of the time and the duration the participant classifies as long 75% of the time. It can be interpreted as a measure of participants’ temporal precision because steep slopes are indicative of precise temporal processing whereas shallow slopes indicate greater variability in the interval-timing system.

ANOVA including factor Group (A *versus* B) and factor Odor (with *versus* without) was performed on PSE and DL.

#### Point of Subjective Equality (PSE)

**FIGURE 1:**
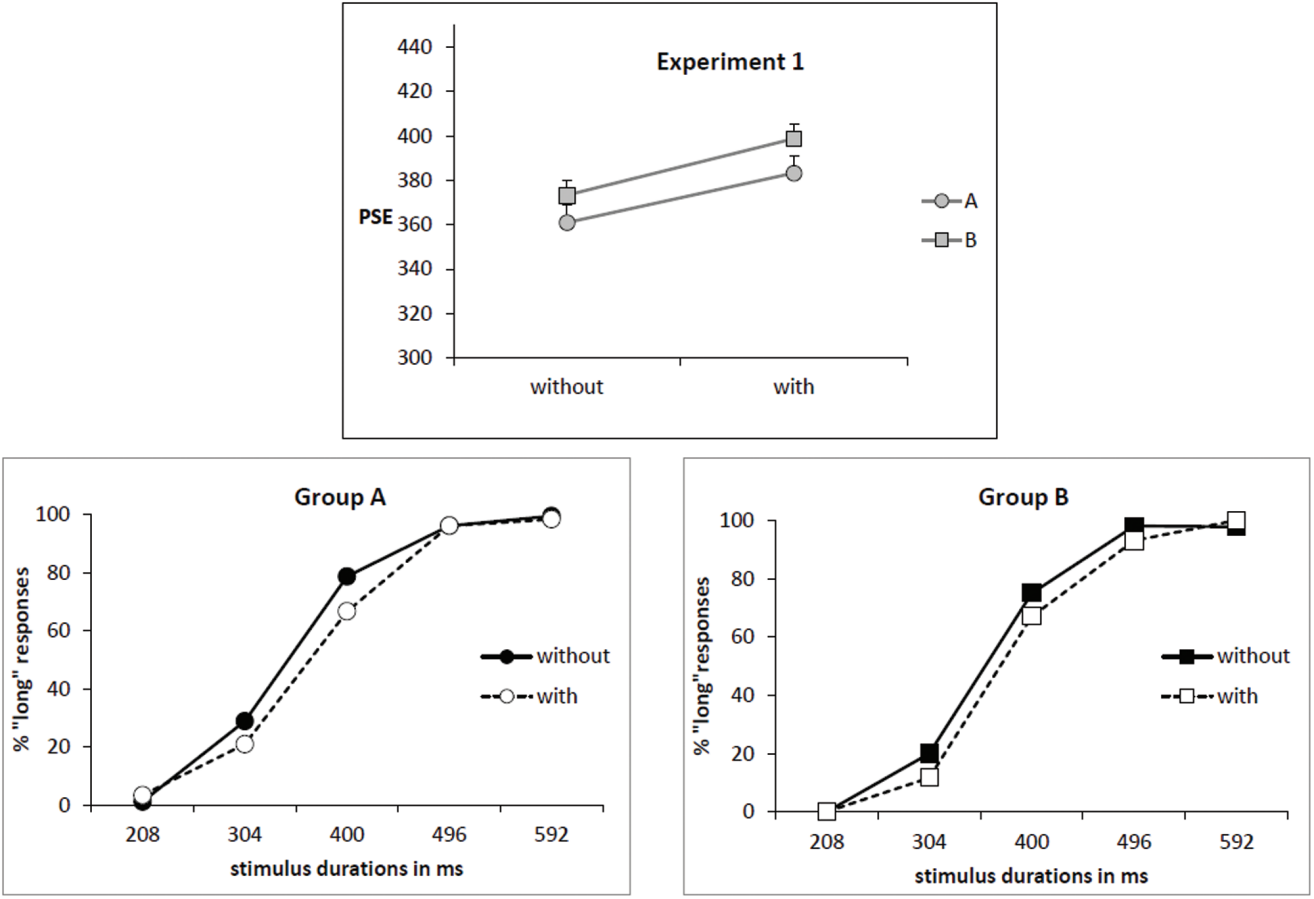
**Experiment 1. (Upper)** Point of subjective equality (PSE in ms) for both groups (A: subjects trained without odor; B: subjects trained with odor) depending on whether they were tested with or without odor. Error bars are standard error of the mean. **(Lower)** Mean proportion of “long” responses plotted against stimulus duration for the two groups of subjects and the two odor conditions.

As illustrated by **Figure 1** (upper part), the mean PSE increased for the condition “with odor” (391 ms) in comparison to the condition “without odor” (367 ms) [*F*_(1,34)_ = 12.07; *p* = 0.001; effect size: Cohen’s *d* = 0.83] and there was no Group × Odor interaction [*F*_(1,34)_ = 0.05; *p* = 0.82]. This corresponds to a rightward shift of the psychometric function in the condition “with odor” as shown on psychometric functions in **Figure 1** (lower part).

The increase of PSE in presence of odor indicates that, when anchor durations were learned without odor (Group A), participants judged intermediate targets as short more often when tested in presence of odor. On the contrary, when participants learned anchor durations in presence of odor (Group B), they judged intermediate targets as long more often when tested without odor compared with when tested with odor (PSE decreased in condition “no odor”). There was no main effect of Group [*F*_(1,34)_ = 1.28; *p* = 0.26]. Moreover, there was no significant correlation between PSE and self-rated values of intensity or hedonic valence.

##### Difference limen (DL)

Concerning the DL (**Table 1**), there was no effect of the odor [*F*_(1,34)_ = 0.2; *p* = 0.65] but variability was larger in Group A (33 ms) compared with Group B (29.8 ms) [*F*_(1,34)_ = 4.34; *p* = 0.04; effect size: Cohen’s *d =* 0.71]. This means that participants who learned anchor durations without odor were more variable in their judgments. There was no significant Group × Odor interaction [*F*_(1,34)_ = 0.77; *p* = 0.38].

**Table 1:** Mean difference limen (DL) for Groups A and B, with or without odor, in Experiments 1 and 2.

	Experiment 1	
	Group A	Group B
Without odor	33.3	28.8
With odor	32.7	30.7

	**Experiment 2**	
	**Group A**	**Group B**

Without odor	128.7	130.4
With odor	133.3	128.9

### Discussion

The aim of the present experiment was to investigate a possible effect of an ambient unpleasant odor on the perception of time by humans. The main result we obtained was that the presence of such an odor produced a shift in temporal judgments towards a shortening of perceived time. Indeed, when participants learned anchor durations without odor and were tested in presence of odor, they underestimated durations. The reverse effect was observed when anchor durations were learned with odor and participants were tested without odor. No effects were observed on variability meaning that time sensitivity was not impaired, as it has already been reported by Droit-Volet et al. (2010) who showed that threatening situations yield time distortions but do not disrupt time discrimination.

In the framework of the pacemaker-counter model, two hypotheses are possible to explain time shortening. The first one is a slowing down of the pacemaker rate. If the clock runs less fast, fewer pulses are accumulated and temporal intervals seem shorter, explaining the rightward shift observed in the PSE. An alternative explanation involves the role of focused attention which has also been pointed out in temporal judgments. It has been proposed that attention may determine the quality of pulse accumulation. Under full attention, the switch is supposed to close and to remain closed for the entire duration of the stimulus whereas, when less attention is being paid, the switch may oscillate or flicker between closed and opened states which would lead to fewer pulses accumulated and then durations judged as shorter, as is the case when a temporal task is made concurrently with an attention-consuming secondary task (Brown, 1997; Casini and Macar, 1997; Burle and Casini, 2001). Effects of emotion of time perception have been explained by modifications of arousal or attention contrasting between positive/neutral and negative hedonic valence (Grondin, 2010; Droit-Volet et al., 2013). Since a slowing down of the pacemaker rate is classically associated with a decrease in arousal level, our data are more consistent with the hypothesis that the unpleasant odor modified the attention level as it has been shown in previous studies (Millot et al., 2002; Michael et al., 2003). In this case, the presence of an unpleasant odor could have captured attention of the participants towards the odor yielding less attention available for temporal processing. This would explain temporal shortening we observed.

Nonetheless, to further investigate a possible effect of arousal on the pacemaker rate, it is interesting to investigate the effect of odor on timing with a different duration range. According to the scalar property, an effect on the pacemaker rate should be multiplicative with the duration values (Penney et al., 2000; Burle and Casini, 2001).

## Experiment 2

The same experimental design was adopted in Experiment 2 except that the durations were centered on 2000 ms.

### Materials and Methods

#### Participants

Thirty-six female undergraduate students (age 18–29 years, mean age = 21.9, *SD* = 2.2) from Besançon University (France) participated into this study. They all were free of nasal allergies and/or head colds and they all gave written informed consent to the experimental procedure. None of these participants took part to Experiment 1.

#### Material and Procedure

The exact same design was used in this experiment except that the anchor durations were 1520 and 2480 ms and the target durations were 1520, 1760, 2000, 2240, and 2480 ms.

### Results

#### Self-Ratings of Odor

The subjects gave the odor a mean rating of 6.05 (*SD* = 1.4) for the perceived intensity and 3.03 (*SD* = 1.72) for the perceived hedonic valence. Data were not significantly different (Student’s *t*-tests) between both experiments, neither for intensity (*t*_70_ = 0.32), nor for hedonicity (*t*_70_ = 0.2).

#### Temporal Task Results

##### Point of subjective equality (PSE)

**FIGURE 2:**
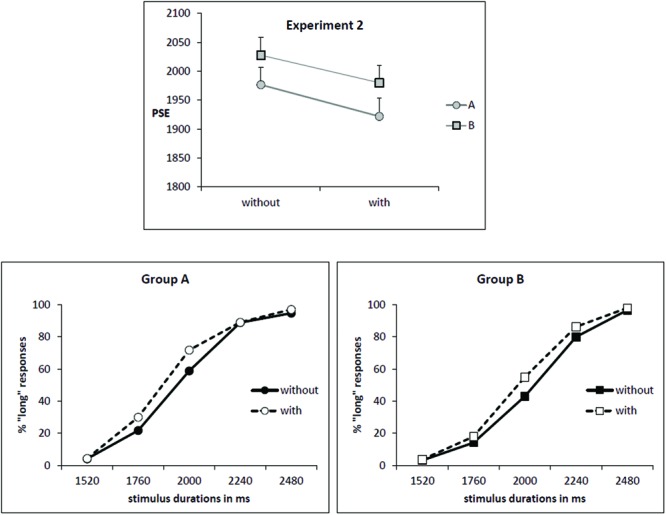
**Experiment 2. (Upper)** Point of subjective equality (in ms) for both groups (A: subjects trained without odor; B: subjects trained with odor) depending on whether they were tested with or without odor. Error bars are standard error of the mean. **(Lower)** Mean proportion of “long” responses plotted against stimulus duration for the two groups of subjects and the two odor conditions.

As illustrated by **Figure 2** (upper part), the mean PSE decreased for the condition “with odor” (1951 ms) in comparison to the condition “without odor” (2002 ms) [*F*_(1,34)_ = 7.36; *p* = 0.01; effect size: Cohen’s *d* = 0.65] and this effect was observed in both groups of subjects (Group × Odor interaction: *F*_(1,34)_ = 0.03; *p* = 0.85]. This corresponds to a leftward shift for the psychometric function in the condition “with odor”, as shown by **Figure 2** (lower part).

The decrease in PSE indicates that, when anchor durations were learned without odor (Group A), participants judged intermediate targets as long more often when tested in presence of odor. On contrary, when participants learned anchor durations in presence of odor (Group B), they judged intermediate targets as shorter more often when tested without odor compared with when tested with odor (PSE increased in condition “no odor”).

An analysis of correlation between PSE and self-rated values revealed that the stronger the subjects perceived the intensity of the odor, the more they judged durations as longer in presence of odor (*r*^2^ = 0.34; *p* = 0.03). There was no significant correlation with hedonic values.

##### Difference limen (DL)

Concerning the DL (**Table 1**), there was no significant main effects [Group: *F*_(1,34)_ = 0.01; *p* = 0.9 and Odor: *F*_(1,34)_ = 0.02; *p* = 0.88], neither a significant Group × Odor interaction [*F*_(1,34)_ = 0.08; *p* = 0.76].

##### Comparison of temporal sensitivity between experiments 1 and 2

To allow for comparing variability through the different duration ranges in Experiments 1 and 2, we computed the Weber fraction (WF) which corresponds to the following ratio DL/PSE for each subject. It is a measure of timing variability that takes into account the duration being timed. The WF are summarized in **Table 2**.

**Table 2:** Mean Weber Fraction (WF) for Groups A and B in each duration range and with or without odor.

	Group A		Group B	
	Short range	Long range	Short range	Long range
WF “without”	0.09	0.06	0.08	0.06
WF “with”	0.09	0.07	0.08	0.06

The data revealed that WF were significantly different depending on the duration ranges [Group A: F_(1,34)_ = 8.41; *p* = 0.007; group B: *F*_(1,34)_ = 7.74; *p* = 0.009]. The variability was significantly larger for the short duration range compared with the long one. There was no significant effect of the odor [Group A: *F*_(1,34)_ = 1.08; *p* = 0.3; group B: *F*_(1,34)_ = 0.00; *p* = 0.99], neither significant Odor x Duration interactions [group A: *F*_(1,34)_ = 0.007; *p* = 0.93; Group B: *F*_(1,34)_ = 0.03; *p* = 0.85].

### Discussion

The main result obtained here is that the unpleasant odor affected time estimation differently in Experiment 2 compared with Experiment 1. While the presence of odor yielded a shortening of time in Experiment 1, here it produced a lengthening of durations.

Lengthening of time is classically explained by an acceleration of the pacemaker rate. When the rate of the internal clock increases, more pulses are accumulated and the signal is perceived to last longer. The main factor thought to be responsible for such an increase is the arousal level (Treisman et al., 1990, 1992; Penton-Voak et al., 1996; Burle and Casini, 2001). In all of these experiments, increasing cortical arousal level with sensory entraining inputs speeds up the rate of the pacemaker.

Interestingly, it has also already been proposed that emotion could modulate the pacemaker rate. Several studies have reported a lengthening of time associated with negative emotion, for example by using angry faces compared with neutral ones (Droit-Volet and Meck, 2007; Gil and Droit-Volet, 2011), threatening signals such as electric shocks or aversive sounds (Droit-Volet et al., 2010), disgusting pictures from IAPS (Gil and Droit-Volet, 2012), or even by showing participants horror films which alter their mood (Droit-Volet et al., 2011). In all cases, time overestimation has been interpreted as an effect of arousal on the internal clock.

Our results suggest that an unpleasant odor would also affect the pacemaker rate. The negative emotion induced by odor would increase arousal and therefore modulate the rate of the internal clock. It is worth noting that the more intense the odor was perceived by subjects, the longer the estimated durations. The arousal level may probably be linked to the perceived intensity of the odor (Bensafi et al., 2002b). Therefore, the results of this second experiment did not confirm the ones of Experiment 1, which was quite unexpected. Nonetheless, they agree with several studies showing time overestimation in the presence of emotion inducer (see Droit-Volet, 2013 for review). They also are in agreement with data recently obtained by Schreuder et al. (2014) in the only study in the literature investigating the effects of odor on perceived duration, at least to our knowledge. The authors have reported that participants exposed to an arousing odor (rosemary) produced significantly shorter time intervals (and thus an overestimation of time perception) than in the no odor condition, which is consistent with an acceleration of the pacemaker rate.

## General Discussion

All the results put together revealed that ambient odor influences time perception but that this effect is different depending on the duration range.

The self-ratings of intensity and of hedonicity were quite similar in both experiments, which mean that the odor was perceived in a similar way in the two situations, with no significant variation in the odor-induced emotional states. We did not assess in this study the level of arousal induced by the odor which is a dimension of odor perception different from the perceived intensity. This perceived intensity was judged as moderate and the pleasantness as negative in both experiments but nonetheless the odor has opposite effect depending on the duration range since it produced underestimation of time for the short duration range and an overestimation for the long one.

Considering the results obtained in the long duration range, they are consistent with several previous studies concluding to an overestimation of time when presenting negative emotional events (Droit-Volet et al., 2004; Gil and Droit-Volet, 2011, 2012). However, unexpectedly, the same odor did not yield the same results in Experiment 1 which involved a short duration range (centered around 400 ms). The underestimation of time observed in Experiment 1 cannot be explained by an increase in arousal but rather by attentional effects. If the unpleasant odor makes subjects focalize their attention on odor instead of on duration of interval, the switch will open and fewer pulses will be accumulated, resulting in a shortening of perceived duration. Lui et al. (2011) recently reported such temporal underestimations in five different experiments. They proposed that their data raised questions about the suitability of internal clock speed explanations of emotion effects on timing and rather highlighted the role of attentional mechanisms. Nonetheless, in our study, it seems that unpleasant odor yields different effects depending on size of the interval to be judged. Along this line, Smith et al. (2011) also reported discrepancies between two duration ranges. In a temporal bisection task using IAPS pictures, they reported an overestimation of intervals with the longest durations and a shortening effect for the shorter durations. They proposed that this shortening effect was due to a rapid activation of the amygdala during the initial perceptual stage (first 300 ms), just before the influence of attentional processing in the extrastriate cortex begins via its connection with the amygdala. Studies on Event-Related Potentials (ERP) have demonstrated that the amygdala influences attention on a specific time scale (Rotschtein et al., 2010). More specifically, the authors found that lesions to the amygdala diminish components of attention at approximately 500–600 ms after the stimulus onset. Although this explanation is speculative and requires further research, it is possible that, in Experiment 1, the unpleasant emotion has attracted attention and triggered a closure of the attentional switch. This very early effect of emotion at the onset of stimulus processing would shorten, rather than lengthen, time estimates. It is difficult to distinguish attention from arousal-related processes, as both seem to play a critical role, and especially since attention and arousal are two distinct but interrelated processes (Paus, 2000). The activation of both attentional and arousal circuits could occur in the brain but may contribute differently along the interval of time. Attentional effects could be predominant at the beginning of the stimulus whereas these initial mechanisms may give way to other processes that modulate arousal levels for longer exposures to stimuli.

An alternative explanation for temporal overestimation, even if less classically considered, could be that participants would voluntarily reinforce their attention towards the duration of intervals in the presence of odor to cancel the effect of the odor as a distractor. They would try to focus their attention on the processing of time more with than without the stimulus. This control would be more difficult when the time discrimination was more difficult in the short duration range, as shown by the larger WF, which could explain the difference of results in the two duration ranges. In the short duration range, it is possible that participants would not have enough time to voluntarily reorient their attention towards the duration of intervals, therefore the only behavioral effect observed would be due to automatic capture of attention by the unpleasant odor.

In future studies, to know whether the temporal overestimation observed in the long duration range would be due to an increase of arousal or to a controlled reorientation of attention towards the duration of the stimulus, a first step could be to require participants to rate the odor on arousal value, which then could be related to the time perception data. Another more sophisticated method would be to evaluate the arousal level trial-by-trial by using psychophysiological measures such as galvanic skin response, heart period, or heart rate variability. On the other side, attentional level could also be manipulated, for example by using dual-task paradigm (see Burle and Casini, 2001) and then the interaction between odor and attentional manipulations could be investigated.

Nonetheless, we cannot exclude that the discrepancy in our results could also come from the two duration ranges used. Indeed, in the field of the psychology of perceived time, a distinction is often made between the processing of durations superior or inferior to one second. In this line, the classical view it that supra-second durations would be cognitively mediated whereas measurement of sub-second durations would be of a highly perceptual nature and not accessible to cognitive control (Rammsayer and Lima, 1991; Lewis and Miall, 2003; Karmakar and Buonomano, 2007).

The analysis of WFs revealed that the presence of odor similarly affected the temporal sensitivity in either of the duration range but a larger temporal sensitivity was observed for the short duration range, indicating a violation of the scalarity. This could support the idea that the processing of the two duration ranges rely on different mechanisms. But it is worth noticing that larger WF for brief durations (inferior to 500 ms) compared to longer ones have already been reported (Getty, 1975; Wearden, 1999). This has been explained by the hypothesis that temporal variability could have two origins, one scalar and one constant. Assuming a source of variance which is not scalar (the variability in the latency of switch opening has sometimes been proposed) would violate the scalarity and yield to larger variability for the shortest durations.

Nonetheless, our results have shown an underestimation of time for the sub-second range and an overestimation for the supra-second range. An explanation would be a differential effect of the olfactory perception on the automatic processing of short durations compared to the cognitive/attention processing needed for long duration processing, but our results have rather suggested an attentional effect for the shortest duration range and therefore do not support the hypothesis of an automatic processing of short durations. But we tested only one unpleasant odor in the present experiments. The results point out the need to test other unpleasant as well as pleasant odors in order to enlarge the conclusions either to olfactory perception and/or to the hedonic valence of the stimulus (emotional states characterized by the pleasure/displeasure dimension). The challenge will be to control the perceived intensity and the lack of trigeminal perception.

To summarize, it is clear that an ambient odor may influence time perception. But, since data led to opposite conclusions in both experiments, the exact mechanisms by which odors influence time perception remain an open question which deserve further investigation.

## Author Contributions

All authors listed, have made substantial, direct, and intellectual contribution to the work, and approved it for publication.

## Conflict of Interest Statement

The authors declare that the research was conducted in the absence of any commercial or financial relationships that could be construed as a potential conflict of interest.
